# Short and long-term impact of intravitreal anti-VEGF therapy interruption in retinal vein occlusion during the COVID-19 pandemic: functional outcomes and AI-based fluid analysis of macular edema

**DOI:** 10.1186/s40942-025-00717-x

**Published:** 2025-08-05

**Authors:** Emmanuelle Moret, Jennifer Cattaneo, Adham Elwakil, Andrea Montesel, Mattia Tommasoni, Chiara M. Eandi

**Affiliations:** 1 Department of Ophthalmology, University of Lausanne, Jules-Gonin Eye Hospital, Fondation Asile des Aveugles, Lausanne, Switzerland; 2 Department of Surgical Science, University of Torino, Torino, Italy; 3 Jules-Gonin Eye Hospital, Avenue de France 15, Lausanne, 1004 Switzerland

**Keywords:** COVID-19, Lockdown, Retina, Intravitreal injections, anti-VEGF, Retinal vein occlusion, Macular edema

## Abstract

**Background:**

The aim of the study is to investigate the short- and long-term effects of delayed intravitreal anti-VEGF injections (IVI) for macular edema (ME) in retinal vein occlusion (RVO) patients during the first wave of the COVID-19 pandemic.

**Methods:**

This is a retrospective observational study analyzing a cohort of patients followed at the medical retina department of the Jules Gonin Eye Hospital. During the COVID-19 lockdown, treatment for patients with ME secondary to RVO was deferred due to emergency federal dispositions. The impact on best corrected visual acuity (BCVA) and on OCT changes (i.e. central subfield thickness (CST), intraretinal fluid (IRF), and subretinal fluid (SRF)) were assessed at several time points before and after the lockdown over a 2-year period. The OCT parameters were assessed by the mean of an artificial intelligence (AI) software (Discovery, RetinAI).

**Results:**

A total of 64 patients were included in the study. BCVA significantly decreased following a mean treatment delay of 10 weeks. However, BCVA returned to baseline levels after 6 months, with no significant differences observed after 2-years of follow-up. OCT analysis revealed an increase in CST, IRF and SRF following the treatment delay, which decreased and return to pre-lockdown values after 3 months. No significant differences in OCT parameters were observed at the two-year follow-up.

**Conclusion:**

The results of our study suggest that delaying IVI for RVO patients during the COVID-19 lockdown resulted in a temporary decline in BCVA and a recurrence of ME. However, these effects were not sustained long term, as both BCVA and ME control returned to baseline levels by 6 months, with no significant changes observed at the two-year follow-up.

**Supplementary Information:**

The online version contains supplementary material available at 10.1186/s40942-025-00717-x.

## Background

Retinal vein occlusion (RVO) is a common vascular disorder affecting the retina. It represents the second leading cause of blindness attributed to retinal vascular diseases, after diabetic retinopathy [1]. RVO can be divided into two main types, according to the anatomic location of the occlusion: branch retinal vein occlusion (BRVO), and central and hemiretinal vein occlusion (CRVO). In addition to this classification, RVOs is further distinguished into ischemic or non-ischemic [2].

Macular edema (ME) is the principal contributor to vision loss in RVO patients [3]. Notably, the resolution of ME in non-ischemic RVO occurs spontaneously in only approximately 30% of cases, with ischemic cases often lacking resolution [4]. In daily clinical practice, ME is assessed and monitored by optical coherence tomography (OCT), which is the method of choice given its precision, non-invasiveness, and safety [5].

Intravitreal anti-VEGF injection (IVI) is the first line treatment for ME secondary to RVO [6–8]. Anti-VEGF agents target VEGF-A, a key molecule involved in intraocular angiogenesis and increased vascular permeability. During the COVID-19 pandemic, aflibercept and ranibizumab were commonly used anti-VEGF agents for ME secondary to RVO. Ranibizumab is a small recombinant fragment of a humanized monoclonal antibody (IgG1 kappa). It consists only of the Fab (antigen-binding) portion, without the Fc (crystallizable) region. It neutralizes all isoforms of VEGF-A. Aflibercept, on the other hand, is a larger fusion protein, composed by the second binding domain of the native VEGF receptor 1 and the third binding domain of the VEGF receptor 2 to the Fc portion of human IgG1. Unlike ranibizumab, aflibercept does not have a Fab region. It has a higher binding affinity for all VEGF isoforms than the native receptors, including VEGF-A, VEGF-B, and PlGF [9–11]. Different anti-VEGF treatment regimens for ME associated with RVO exist. Common approaches include monthly injections over 3 months, followed by a pro re nata (PRN) approach, or a treat-and-extend (T&E) regimen tailored to disease activity [6, 7, 12, 13].

The advent of the COVID-19 pandemic prompted significant adjustments in medical practices to reduce disease exposure risks. Governments worldwide implemented restrictive measures to safeguard public health and prevent healthcare system overload. In Switzerland, a partial lockdown period from March 16 to April 27, 2020, led to the deferral of non-urgent medical consultations and procedures.

In the field of ophthalmology, adaptations were necessary. Collective recommendations, including those for intravitreal anti-VEGF treatment, were issued by scientific societies such as the Vision Academy Steering Committee [14]. Consequently, most of RVO patients with ME had deferred or suspended treatment protocols during the lockdown, which gradually return to normal after restrictions were lifted.

Artificial Intelligence (AI) has shown significant promise in ophthalmology. It enhances the detection, diagnosis, and monitoring of retinal diseases (e.g. Age-related Macular Degeneration (AMD), Diabetic Retinopathy, Retinopathy of Prematurity). AI applied to OCT imaging enables precise evaluation of OCT-derived features, including central subfield thickness (CST), as well as the quantification of intraretinal (IRF) and subretinal fluids (SRF) through automated fluid segmentation and volume calculation [15].

This study aims to investigate the short and long-term effects, up to 2 years, of delayed anti-VEGF IVI for ME in RVO patients during the first wave of COVID-19 in a Swiss referral center. We evaluated both functional (BCVA) and anatomical outcomes (OCT), with the latter analyzed using AI with a combination of the RetinAI Discovery software [16] and of our in-house software pipeline Cohort Builder [17].

## Methods

The study received approval from the local institutional review board and ethical committee (CER-VD n. 2021 − 01612) and adhered to the principles of the Declaration of Helsinki. All patients had given written informed consent.

### Lockdown management

During spring 2020, the Swiss government enforced the postponement of non-urgent interventions to curb the spread of COVID-19 [18]. A partial lockdown was implemented from March 16th to April 27th, 2020. Throughout this period, at the medical retina department of Jules Gonin Eye Hospital – which is a tertiary referral center – all routine outpatient consultations were suspended, with only urgent therapy being maintained. To assess the urgency of each case, patients were individually evaluated to determine the visual risk in the event of IVI postponement.

Guidelines from ophthalmological societies and experts’ recommendations were followed to decide for postponing or proceeding with injections. Patients affected by RVO were considered as a “low” priority class, and consequently, their IVI treatments were deferred, as they were less likely to experience irreversible vision loss in the short term [19]. An exception was made for RVO patients who met specific “high-risk” criteria for vision-threatening complications, defined as being in the acute phase of RVO (within the initial loading period of less than 3 months) or having only one functional eye. All these considerations were carefully balanced with the patient’s overall health status and personal preferences – particularly the fear of leaving home to receive treatment – which influenced the decision to either defer or proceed with the IVI. If IVI was maintained, it occurred without attending consultations or undergoing OCT, following an established “injection-only approach” as recommended by the French Society of Ophthalmology guidelines for managing patients receiving IVI during the COVID-19 pandemic [14, 19, 20].

### Study design and participants

In this retrospective observational monocentric cohort study, we enrolled all patients scheduled for IVI for ME secondary to RVO during the lockdown period. Eligible patients had a confirmed diagnosis of ME secondary to RVO prior to the lockdown, had undergone at least one clinical evaluation with OCT imaging, and had received at least one IVI before the lockdown. Consequently, patients with newly diagnosed ME due to RVO during the lockdown – i.e., treatment-naïve eyes – were not included in the study.

In our department, the standard treatment for patients with ME secondary to RVO is a loading dose of 4 monthly intravitreal injections of ranibizumab or aflibercept 2 mg, as per label, followed by a T&E regimen with a 2-weeks extension interval.

Nine time points were chosen for the study: three preceding the lockdown (T-3, T-2, T-1), and five following it (T1, T2, T3, T4, T5). Baseline was defined as the date of scheduled IVI during the lockdown period. T-1 represented the last visit before Baseline, while T-2 and T-3 were set at 3 and 6 months before T-1, respectively. T1 was defined as the first visit after the lockdown and T2, T3, T4, T5 respectively corresponded to 3, 6, 12, and 24 months after T1. Patients were required to have records for at least T-1, T1, and T2 to be included in the study.

Exclusion criteria were the presence of maculopathy from other causes than RVO, and a history of vitreomacular surgery during the study period.

At baseline, patient demographics, eye side, cataract status, the type of RVO (branch or central) and the presence of ischemia, the date of diagnosis, the number of previous IVIs in the preceding 3 and 6 months before the last visit prior to lockdown, the last interval between IVI, as well as the type of anti-VEGF medication used were collected. The diagnosis of RVO, along with its ischemic status, had been previously established by a retinal specialist and documented in the patient’s medical record before the start of the study. Fluorescein angiographies were also reviewed during data collection to confirm the presence or absence of ischemia. In case where the presence of ischemia was uncertain, the final decision was made by an experienced retinal specialist (C.M.E.).

Best-corrected visual acuity, routinely measured with patient refraction on an early treatment diabetic retinopathy study (ETDRS) chart and expressed as ETDRS Letter Score for statistical analysis, was documented at each time point (T-3 to T5).

### OCT analysis

All patients had routine macular volume scan with spectral-domain (SD) OCT (Spectralis^®^ HRA-OCT, Heidelberg Engineering, Heidelberg, Germany) at each time point.

Spectral-domain OCT volume scans (macular map, 30 × 30, 10 ART, 49 scans) were subsequently imported into the RetinAI Discovery software [16]. This AI-based platform utilizes deep learning algorithms trained to replicate expert-level segmentation of OCT-derived features. The extracted features included CST within the 1-mm central subfield of the ETDRS grid, as well as automated quantification of IRF and SRF through fluid segmentation and volume calculation across the nine ETDRS subfields for each OCT volume scan, following previously validated methodologies in neovascular AMD [21]. Data extraction was performed using an in-house software pipeline, Cohort Builder [17]. The process involved inputting a list of patient identifiers based on diagnosis into Cohort Builder, which verified authorization through the hospital’s General Consent database. Upon verification, the software retrieved the raw fovea-centered OCT imaging dataset and uploaded it into the Discovery platform via its Application Programming Interface (API). Discovery then processed the images, extracting OCT-derived features of interest. Finally, Cohort Builder retrieved these data in tabular format, ready for further analysis.

Macular edema was defined as the presence of IRF and/or SRF on OCT macular volume scan. A worsening in ME corresponded to an increase in IRF and/or SRF, while an improvement was defined as a reduction in IRF and/or SRF. ME was considered stable when no changes in IRF and/or SRF were observed.

OCT scans quality was manually assessed before automatized calculation by two independent and experienced readers (E.M. and J.C), ensuring the foveal centering and the fluid segmentation were correct. Scans that were not well centered or segmented, as well as the ones with insufficient quality for identification of fluid or CST were excluded from the analysis. In case where the graders did not agree on a single consensus result, the disagreement was resolved by a third experienced retinal specialist (C.M.E.).

### Primary and secondary endpoints

The study aimed to assess whether the delay in IVI had any short- or long-term effects on visual function or the anatomical structure of the macula in patients with ME secondary to RVO. The primary endpoint was the comparison of BCVA and OCT parameters (i.e. CST, IRF, and SRF) before the lockdown with those obtained after, and during the two years following the resumption of normal medical activities.

Additionally, the secondary endpoints were to compare these changes across different subgroups, specifically drug type (aflibercept, ranibizumab), eye side, type of RVO (central or branch, ischemic or non-ischemic), type of fluid at T-1 / T1 (IRF, SRF), and ME duration (recent vs. long-standing). In this study, recent ME was defined as a duration of less than 6 months since diagnosis and included patients in the acute phase of RVO, i.e. those within the initial loading period (less than 3 months). Long-standing ME was defined as lasting more than 6 months since diagnosis.

### Statistical analysis

Data were collected and statistically analyzed using Python, employing the following libraries: pandas (library version 1.5.3), numpy (library version 1.24.0), scipy (library version 1.9.3), statsmodels (library version 0.13.5), matplotlib (library version 3.6.3), and seaborn (library version 0.11.2) for data processing, statistical testing, and visualization. Visualization of results was carried out using matplotlib (library version 3.6.3) and seaborn (library version 0.11.2).

Continuous variables are presented as mean ± standard deviation (SD) for normally distributed data, and as median (interquartile range, IQR) for non-normally distributed data, while categorical variables are expressed as frequencies and percentages. The Shapiro-Wilk test was used to assess normality of continuous variables. Depending on normality assumptions, parametric tests (e.g., t-test) or non-parametric tests (e.g., Wilcoxon signed-rank test, Mann-Whitney U test) were applied for comparisons between groups. Homogeneity of variances was evaluated using Levene’s, Bartlett’s, and Fligner-Killeen tests. For analysis of variance, one-way ANOVA was performed, with post-hoc comparisons as appropriate. Statistical significance was set at *P* < 0.05.

Data preprocessing involved calculating time differences in months to support longitudinal analyses. To ensure consistency in statistical modeling, patient data were systematically indexed and arranged in chronological order. The time interval between consecutive visits or events was determined by computing the absolute difference in months between two given dates. This approach facilitated the precise alignment of follow-up measurements across patients, enhancing the reliability of temporal analyses.

## Results

### Patients’ characteristics

**Fig. 1:**
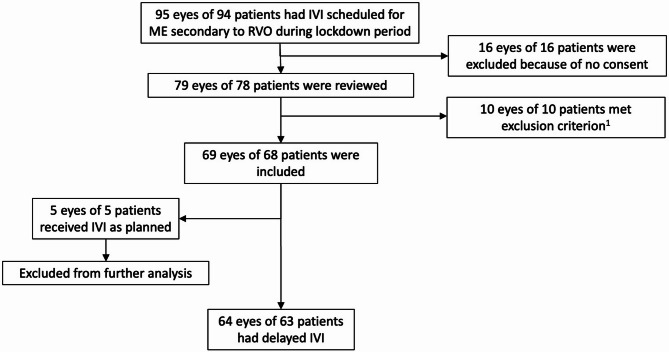
Flow-chart of included patients with IVI scheduled for ME secondary to RVO. ^1^ exclusion criterion: other macular disease, lost in follow up, history of vitreomacular surgery. IVI: anti-VEGF intravitreal injection; ME: macular edema; RVO: retinal vein occlusion

During the lockdown period, 95 eyes of 94 patients were scheduled to receive anti-VEGF IVI for ME secondary to RVO. 16 patients were excluded due to lack of consent for research. Additionally, 4 eyes of 4 patients were excluded due to concomitant diagnose of retinal disease that could affect the results (type 1 macular telangiectasia, age-related macular degeneration, occlusive retinal microvasculopathy with positive HLA-B51 and ANA, vasculitis, vitreomacular traction). Furthermore, 4 eyes of 4 patients were lost to follow-up before T2. Two eyes of 2 patients underwent vitreomacular surgery for epiretinal membrane peeling during the follow-up period. Ultimately, a total of 69 eyes of 68 patients were included in the study (Fig. 1).

Patients who received an IVI at baseline were excluded from further analysis (Fig. 1). This applied to only 5 out of the 69 included eyes: two were administered due to a recent diagnosis of ME secondary to RVO within the past month (loading dose), two were given due to decreased vision lasting less than 10 days with a confirmed increase in ME on OCT, and one was administered because the patient refused to discontinue IVI despite medical advice.

Table 1 provides a summary of the baseline characteristics of the study population – including gender, age, eye side, type of RVO (central vs. branch), ischemic status, recent or long-lasting ME, and drug type (ranibizumab vs. aflibercept).

**Table 1:** Characteristics of the study population

	*n* = 64
**Gender** Female (%) Male (%)	35 (54.69%) 29 (45.31%)
Mean age (years old) ± SD (range)	75.94 ± 12.22 (63.72–88.16)
**Eye side** RE (%) LE (%)	29 (45.31%) 35 (54.69%)
**RVO** Central Branch	35 (54.69%) 29 (45.31%)
**Ischemic** Yes No	35 (54.69%) 29 (45.31%)
**Diagnosis of ME** <6months (“recent”) >6 months (“long-lasting”)	7 (10.94%) 57 (89.06%)
**Drug** Ranibizumab Aflibercept	37 (57.81%) 27 (42.19%)

RE: right eye; LE: left eye; RVO: retinal vein occlusion; ME: macular edema; SD: standard deviation

The median time points for T-3, T-2, T-1, T1, T2, T3, T4, and T5 are recorded in Table 2.

**Table 2:** Median times points (from T-3 to T5) and follow up rate

	T-3	T-2	T-1	B	T1	T2	T3	T4	T5
Total No. of studied eyes (%)	55 (85,9%)	62 (96,9%)	64 (100%)	64 (100%)	64 (100%)	64 (100%)	62 (96,9%)	59 (92,2%)	54 (84,4%)
Months Median (IQR) (range 25th 75th ) (range min max)	-8.07 (1.93) (-9.18 to -7.25) (-14.47 to -5.33)	-4.65 (1.82) (-5.76 to -3.94) (-11.20 to -3.03)	-1.95 (1.21) (-2.80 to -1.59) (-5.60 to 0.00)	0	+ 2.57 (1.07) (+ 1.96 to + 3.03) (0.00 to + 5.63)	+ 5.60 (1.44) (+ 4.87 to + 6.31) (+ 3.20 to + 9.30)	+ 9.10 (1.93) (+ 8.17 to + 10.09) (+ 6.03 to + 13.53)	+ 15.27 (2.00) (+ 14.33 to + 16.33) (+ 12.10 to + 19.93)	+ 27.45 (2.70) (+ 26.62 to + 29.32) (+ 25.40 to + 31.53

IQR: interquartile range

Over the follow-up a few subjects were lost for two main reasons, either they moved to a different clinic closer to their domicile or they passed away. In particular, 96,9% of the eyes (62 out of 64 eyes) were tracked up to T3, 92,2% (59 out of 64 eyes) up to T4, and 84.4% (54 out of 64 eyes) up to T5 (Table 2).

### Visual acuity

Before the lockdown, the median BCVA for cohort was 80 ETDRS letters at T-1. After resuming clinical activities post-lockdown, median BCVA significantly decreased to 75 ETDRS letters at T1 (Table 3a), while by T3 median BCVA recovered to same pre-lockdown levels, at 80 ETDRS letters.

**Table 3a:** Median BCVA, CST, IRF and SRF volume at each time points for the overall population

	BCVA	CST (µm)	IRF (nl)	SRF (nl)
T-3 Median (IQR) (range 25th 75th) (range min max)	80.00 (15.00) (70.00–85.00) (40.00–90.00)	280.03 (55.54) (257.68-313.22) (197.14-372.42)	35.29 (226.76) (3.17-229.93) 0.00-2133.46	0.00 (0.11) (0.00-0.11) (0.00-480.79)
T-2 Median (IQR) (range 25th 75th) (range min max)	75.00 (20.00) (65.00–85.00) (0.00–90.00)	288.90 (61.03) (263.69-324.72) (172.70-460.57)	44.20 (257.93) (1.51-259.45) (0.00-3737.43)	0.00 (0.16) (0.00-0.16) (0.00-342.62)
T-1 Median (IQR) (range 25th 75th) (range min max)	80.00 (20.00) (65.00–85.00) (35.00–95.00)	279.57 (62.53) (255.75-318.27) (179.31-406.65)	24.28 (191.50) (0.89-192.39) (0.00-5181.17)	0.00 (0.21) (0.00-0.21) (0.00-2449.96)
T1 Median (IQR) (range 25th 75th) (range min max)	75.00 (26.25) (58.75-85.00) (0.00–90.00)	304.74 (71.53) (271.03-342.56) (180.69–565.40)	185.68 (883.50) (25.56-909.06) (0.00-6331.10)	0.00 (3.15) (0.00-3.15) (0.00-3274.20)
T2 Median (IQR) (range 25th 75th) (range min max)	75.00 (20.00) (65.00–85.00) (0.00–95.00)	271.50 (60.21) (240.91-301.12) (177.70-415.55)	5.14 (65.50) (0.23–65.73) (0.00-3197.62)	0.00 (0.11) (0.00-0.11) (0.00-116.86)
T3 Median (IQR) (range 25th 75th) (range min max)	80.00 (18.75) (66.25-85.00) (35.00–90.00)	285.24 (63.95) (246.78-310.73) (181.73-412.84)	16.62 (141.77) (0.85-142.62) (0.00-4011.04)	0.00 (0.13) (0.00-0.13) (0.00-3739.84)
T4 Median (IQR) (range 25th 75th) (range min max)	80.00 (20.00) (65.00–85.00) (0.00–90.00)	272.98 (58.16) (252.45-310.61) (165.03–440.00)	8.85 (83.57) (0.31–83.88) (0.00-2977.19)	0.00 (0.25) (0.00-0.25) (0.00-85.08)
T5 Median (IQR) (range 25th 75th) (range min max)	80.00 (15.00) (70.00–85.00) (0.00–95.00)	275.29 (58.25) (247.43-305.68) (165.28–458.00)	5.33 (63.02) (0.33–63.35) (0.00-4859.26)	0.00 (0.01) (0.00-0.01) (0.00-685.86)

BCVA: best corrected visual acuity; CST: central subfield thickness; IRF: intraretinal fluid; SRF: subretinal fluid; IQR: interquartile range

Supplementary graphic 1 illustrates the changes in median BCVA across all time points. Median changes in BCVA values for each visit compared to T-1 are detailed in Table 3b, with statistically decrease in BCVA at T1 compared to T-1. No statistically significant differences in BCVA were observed at the follow-up visits T3, T4, and T5.

**Table 3b:** Median changes in BCVA, CST, IRF, and SRF compared to T-1. For the overall population

	Change in VA compared to T-1	*p*-value	Change in CST compared to T-1	*p*-value	Change in IRF compared to T-1	*p*-value	Change in SRF compared to T-1	*p*-value
T-3 Median (IQR) (range 25th 75th) (range min max)	0 (0) (0.0–0.0) (0.0–5.0)	0.9834	4.71 (8.17) (3.50-11.67) (17.83–7.56)	0.0648	18.60 (62.17) (2.42–64.59) (0.00-3047.71)	0.1358	0 (0.02) (0.00-0.02) (0.00-332.45)	0.9234
T-2 Median (IQR) (range 25th 75th) (range min max)	-5 (1.25) (-1.25-0.0) (-35.0-5.0)	0.0988	10.11 (9.92) (8.44–18.36) (-6.62-60.57)	0.0383	25.27 (88.44) (0.65–89.09) (0.00-1443.74)	0.2600	0 (0.05) (0.00-0.05) (0.00-186.66)	0.2640
T1 Median (IQR) (range 25th 75th) (range min max)	-5 (6.25) (-6.25-0.0) (-35.0-5.0)	0.0005*	25.17 (9.01) (15.28–24.29) (1.37-158.75)	0.0000*	161.40 (692.00) (24.67-716.66) (0.00-1149.93)	0.0000*	0.0015 (2.94) (0.00-2.94) (0.00-824.23)	0.0084*
T2 Median (IQR) (range 25th 75th) (range min max)	-5 (0) (0.0–0.0) (-35.0-0.0)	0.2330	-8.07 (-2.31) (-14.84-17.15) (-1.62-8.90)	0.4379	-19.13 (-126.00) (-0.66-126.66) (0.00-1983.55)	0.0369*	0 (-0.10) (0.00-0.10) (0.00-2333.10)	0.3779
T3 Median (IQR) (range 25th 75th) (range min max)	0 (3.75) (-3.75-0.0) (0.0–5.0)	0.5383	4.68 (-0.60) (-14.09-14.69) (2.41–6.20)	0.3350	-2.30 (-51.85) (-0.01-51.87) (0.00-1170.13)	0.2804	0 (-0.14) (0.00-0.14) (0.00-1289.87)	0.2556
T4 Median (IQR) (range 25th 75th) (range min max)	0 (2.50) (-2.5-0.0) (-35.0-5.0)	0.0424*	-6.00 (-5.94) (-5.30-11.24) (-14.28-33.35)	0.2672	-7.84 (-112.20) (-0.57-112.77) (0.00-2203.98)	0.7690	0 (0.10) (0.00-0.10) (0.00-2364.88)	0.5666
T5 Median (IQR) (range 25th 75th) (range min max)	0 (0) (0.0–0.0) (-35.0-0.0)	0.3877	-3.69 (-11.33) (-13.09-24.42) (-14.03-51.35)	0.0662	-15.84 (-131.61) (-0.58-132.19) (0.00-321.91)	0.0414*	0 (-0.10) (0.00-0.10) (0.00-1764.10)	0.0199*

*Statistically significant *p* < 0.05. BCVA: best corrected visual acuity; CST: central subfield thickness; IRF: intraretinal fluid; SRF: subretinal fluid; IQR: interquartile range

When analyzing the different subgroups, patients with RVO and recent ME had a significant difference in BCVA at T-2 and T-1 (additional Table 1). However, the post-lockdown evolution of BCVA did not differ significantly between patients with recent or long-standing ME. Specifically, no significant difference in BCVA was observed at T5. Central RVO had significant worse BCVA from T-2 to T4 compared to BRVO. Ischemic RVO experienced a greater decline in BCVA than non-ischemic RVO at T1, but this difference was not observed at later time points. Unsurprisingly, patients with IRF at T1 had significantly worse BCVA compared to those without IRF at the same time point. However, this difference was not observed at subsequent time points when IRF resolved. Additionally, no significant differences in BCVA were observed based on the type of fluid at T-1 (e.g., SRF vs. IRF) or the anti-VEGF drug used (ranibizumab vs. aflibercept) (additional Table 1).

### OCT findings and ME recurrence after lockdown

**Fig. 2:**
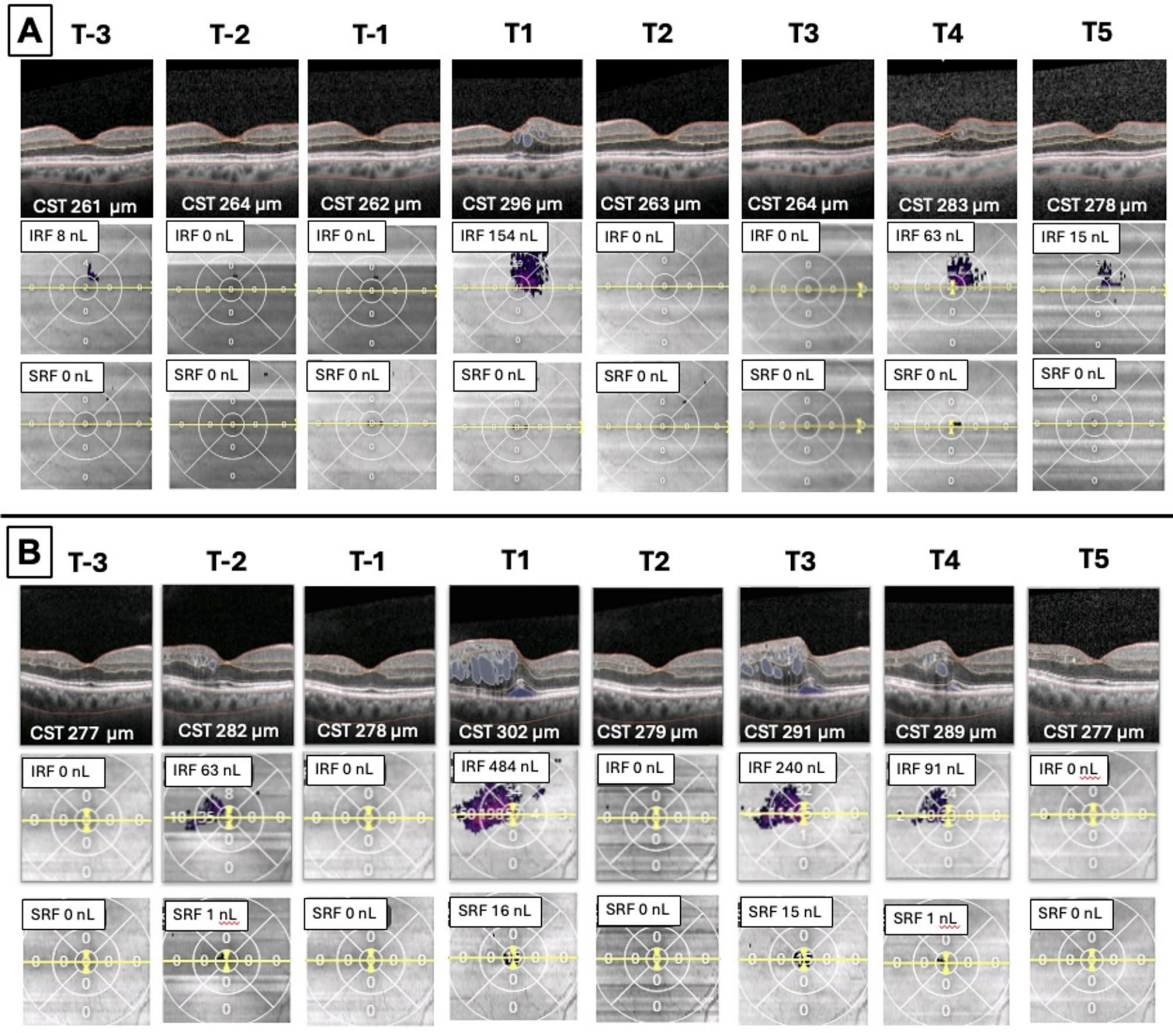
**A** and **B**. Representative cases at the different time points. IRF: intraretinal fluid. SRF: subretinal fluid

The increase in ME was associated with an elevation in CST and an increase in the volume of IRF and SRF (see representative cases in Fig. 2). Additional graphic 2 illustrates the variation in CST, while Additional graphic 3 shows the changes in IRF and SRF. For the overall population, we observed an increase in CST, IRF and SRF at T1, which then decreased at T2. There was no significant difference in CST and the amount of SRF at T2 compared to T-1. IRF amount was even significantly lower in T2 compared to T-1 (Table 3b).

OCT findings were compared across the different subgroups (additional Table 1). The results showed a greater increase in CST, IRF and SRF at T1, which returned to pre-lockdown values by T2, with no statistically significant differences related to drug type or treated eye side. In ischemic RVO compared to non-ischemic RVO, there was a greater increase in CST and IRF in ischemic cases at T1, but this difference was not statistically significant at T5. Similarly, CRVO showed a greater increase in these parameters compared to BRVO, though without statistical significance at T5. Among patients with RVO and ME, there was a higher volume of fluid at T-1 and T-2 in recent ME compared to long-standing ME. However, this difference disappeared after the lockdown period, particularly over the long term at T5.

### IVI characteristics and subsequent treatments

The IVI characteristics are detailed at each time point (i.e. number of IVI between time points and IVI interval) for all patients in Table 4. The median number of IVI during the 6 months period (from T-3 to B) before the lockdown was 4 (IQR 2; range quantile 3–5), while patients received a median of 2, 2, 3, 5 IVI 3, 6, 12, and 24 months after the lockdown, with a median of 12 IVI (range quantile 5–16) over the 2 years since the resuming of the clinical activity. Among the 64 eyes, 11 (17.2%) did not receive additional IVI following the resumption of medical activities after lockdown: 3 eyes because of refractory chronic ME and 8 eyes showing no recurrence of ME. The proportion of patients observed without further IVIs increased over time: 17.2% at T1, 20.3% at T2, 24.2% at T3, 25.4% at T4, and 37.5% at T5 (Table 4).

**Table 4:** IVI characteristics at different time points for the overall population

	No. of treated eyes (%)		Overall (IQR) (range 25–75 quantile) (range min max)
No. of IVI Median (IQR)	64 (100%)	From T-3 to B	4.00 (2.00) (3.00–5.00) (0.00–6.00)
	53 (82.8%)	From B to T1	0.00 (0.00) (0.00–0.00) (0.00–12.00)
	51 (79.7%)	From T1 to T2	2.00 (2.00) (1.00–3.00) (0.00–4.00)
	47 (75.8%)	From T2 to T3	2.00 (2.00) (1.00–3.00) (0.00–4.00)
	44 (74.6%)	From T3 to T4	3.00 (3.50) (0.50–4.00) (0.00–7.00)
	35 (62.5%)	From T4 to T5	5.00 (7.00) (0.00–7.00) (0.00–12.00)
	35 (62.5%)	From T1 to T5	12.00 (11.00) (5.00–16.00) (0.00–24.00)
IVI interval (weeks) Median (IQR)		Before B	6.00 (4.00) (4.00–8.0) (4.0–12.0)
		at T2	6.00 (3.50) (4.50-8.0) (4.0–16.0)
		at T3	8.00 (2.00) (6.00–8.0) (4.0–14.0)
		at T4	7.50 (4.00) (6.00–10.0) (4.0–14.0)
		at T5	8.00 (4.00) (6.00–10.0) (4.0–22.0)
T-1 – T1 (cessation treatment) Median (IQR)			17.00 (6.14) (13.75–19.89) (5.14–32.43)
Delay Median (IQR)			10.00 (4.86) (8.07–12.93) (0.00-25.43)

IVI: intravitreal anti-VEGF injections; IQR: Interquartile range

The overall median interval between IVI before the lockdown was 6 weeks and remained unchanged during the first three months after the treatment was restarted (at T2), while it then increased over the follow-up to 8, 7.5 and 8 weeks at 6, 12, and 24 months (T3, T4 and T5), respectively.

For the 64 eyes of 63 patients who received IVI after the lockdown, the median delay was 10 weeks (Table 4).

## Discussion

The COVID-19 pandemic significantly disrupted ophthalmic practices worldwide due to government-imposed lockdowns and stay-at-home orders [14, 18–20]. During this time, medical care was limited to emergencies, leading to the suspension of elective procedures, including IVI. This situation particularly affected patients with ME secondary to RVO, as their scheduled treatments were mostly postponed according to international expert recommendations [14]. Indeed, RVO patients were classified as a “low” priority group, given the low risk of irreversible vision loss in the short term due to delayed IVIs [19].

Our results demonstrated that delays in care were associated to short-term vision loss. Nevertheless, BCVA returned to pre-lockdown levels within six months of resuming regular treatment and remained stable over the long term, up to two years of follow-up. These findings are consistent with previous studies that reported initial vision deterioration after delayed IVIs [22, 23]. Song et al. showed no significant difference in visual acuity compared to pre-lockdown levels after one year of follow-up (with an average IVI delay of 5.7 weeks) [24]. Our study showed similar results, with a median delay of 10 weeks.

Notably, the results were consistent across subgroups (eye side, branch vs. central, ischemic vs. non ischemic, drug type, and type of fluid), with vision returning to pre-lockdown levels and remaining stable for up to two years. Interestingly, when comparing patients with recent ME (< 6 months) to those with long-standing ME (> 6 months), after two years, BCVA were statistically equivalent to those in both groups. This suggests that the delay in IVI during or near the loading dose phase did not result in significant vision loss. We also observed that eyes with CRVO experienced worse initial visual impairment compared to those with BRVO. This difference arises from the distinct characteristics of each condition. In both cases, ME results from multifactorial pathophysiological changes, including breakdown of the blood–retinal barrier due to impaired capillary flow. This disruption alters the balance of angiogenic and inflammatory cytokines in the ocular fluid. Studies show that levels of VEGF-A and inflammatory markers like interleukin-6 (IL-6) are higher in undiluted vitreous samples of CRVO eyes than in BRVO eyes. These higher levels are linked to more severe ME. As a result, CRVO causes a stronger inflammatory and growth response, leading to more severe ME and, consequently, greater vision loss. Furthermore, the more extensive retinal damage in CRVO contribute to this disparity [25–27].

Early anti-VEGF treatment is generally recommended for RVO. Although some studies have demonstrated that delayed therapy can still lead to improvements in BCVA, these gains are typically less pronounced than those observed with early intervention [28–30]. The CRYSTAL study demonstrated that visual outcomes were significantly better in patients who received treatment within 3 months of diagnosis compared to those treated after 3 months [31]. Similarly, the COPERNICUS study evaluated the efficacy of intravitreal aflibercept in CRVO and found that visual prognosis worsened and irreversible retinal damage occurred when treatment was initiated more than 6 months after symptom onset. Furthermore, the visual prognosis of patients who started treatment within 2 months was better than those who initiated treatment after 2 months [32]. In line with these findings, the RVO guidelines in 2022 suggested that time to initial evaluation and treatment should occur within 2–4 weeks of symptom presentation, with treatment initiated within 1–2 weeks of assessment in appropriate facilities for intravitreal injection [33]. Patients who began treatment 28 days or more after symptom onset experienced a smaller improvement in VA from their first visit to the initiation of therapy, compared to those treated within 28 days. Similarly, patients treated after 15 to 28 days had worse VA outcomes than those treated within 14 days. These findings suggest that a delay of 14 to 28 days or more before initiating treatment may be associated with poorer VA at baseline and a less favorable visual prognosis in patients with CRVO [34]. In contrast to these findings, our results suggest that in our cohort, the potential for visual recovery is preserved even when treatment is delayed. This observation is consistent with previous reports – for example, Im et al. observed that delayed treatment (of a mean of up to 4.8 months) did not impact BCVA [35]. Similarly, Liu et al. found no difference in BCVA, although there was a persistent increase in CST [36]. These observations suggest that, in certain cases, a delay in treatment may not significantly compromise visual recovery. This appears particularly true for patients with good baseline visual acuity and no evidence of severe macular ischemia. Even in the presence of prolonged ME, visual function may recover if the ellipsoid zone (EZ) remains intact [37]. Furthermore, unlike diabetic macular edema (DME), ME secondary to RVO tends to cause fewer disorganization to the inner retinal layers (DRIL) [38]. These factors may help explain why visual acuity was able to return to pre-lockdown levels despite delayed treatment.

Central subfield thickness followed the same trend that BCVA. The study by Song et al. did not find an increase in the mean CST at one year compared to pre-lockdown levels, for both patients who received IVI during the lockdown and those who did not. Our findings align with this, as CST returned to pre-lockdown levels within three months after resuming normal medical activity, without difference between subgroups (eye side, branch vs. central, ischemic vs. non ischemic, drug type, and type of fluid).

The volumes of IRF and SRF were quantified using automated segmentation powered by the RetinAI Discovery deep-learning algorithm [21]. This algorithm was developed and trained to identify, localize, and measure pathological exudation in SD-OCT scans, achieving high sensitivity and specificity. It employs a fully automated convolutional neural network (CNN) architecture, incorporating retinal layer segmentation to improve fluid detection and quantification. The method enables volumetric assessment and three-dimensional visualization of fluid compartments, allowing for precise qualitative and quantitative analysis of ME. The same algorithm has been validated for use in neovascular AMD [21], demonstrating strong correlation between automated and expert manual segmentations with high reproducibility across multiple scans. Additionally, it has been applied to DME and RVO [39], reinforcing its potential utility across various retinal pathologies. Fluid analysis offers valuable insights beyond CST alone. While CST provides a quantitative measure limited to fovea-involving ME, fluid volume analysis provides a more comprehensive evaluation by assessing fluid across the entire ETDRS macular grid, including regions where ME does not reach the fovea. We assume that this method is more precise and more sensitive to fluctuations, providing a more accurate estimation of overall ME. Moreover, IRF and SRF are considered predictive factors for visual prognosis [40, 41], and SRF has shown to be predictive of higher disease activity in BRVO [40]. In our study, we did not observe a significant correlation between the presence of IRF/SRF and worse BCVA or ME control after IVI delay in the long term.

We observed that IRF and SRF volumes initially increased at the first post-lockdown visit, corresponding with the recurrence of the ME. However, these volumes decreased within three months of resuming regular treatment. Specifically, IRF levels significantly dropped by the second follow-up visit (T2), reaching values even lower than those recorded before the lockdown. This suggests that the ME was more effectively controlled, likely due to a more aggressive treatment approach in response to the recurrence of ME.

Notably, 17.2% of patients did not need further IVI during the follow-up (either because of unchanged or no ME recurrence after lockdown), suggesting potential anti-VEGF IVI overtreatment with the T&E regimen. For all patients who continued with IVI and T&E regimen, the mean interval between IVI increased over time after resuming regular activity, reaching 8 weeks interval at last follow-up visit at 24 months. This progressive increase in intervals is consistent with the one published in the T&E protocol for CRVO, which showed longer intervals over time and a consequent reduction in the number of IVIs per year in the long term [13]. This suggests that the increase in intervals with time was not adversely affected by the delay in treatment during lockdown.

The main limitations of our study are its retrospective design and the relatively small sample size. However, its strengths include the two-year follow-up period and the use of deep-learning artificial intelligence for precise quantitative measurement of retinal fluids. A further limitation of this study is that the AI platform used does not support the qualitative assessment of key structural biomarkers —such as DRIL, EZ integrity, and external limiting membrane continuity — which are well-established predictors of visual function but were not evaluated in this analysis.

## Conclusion

The results of our study indicate that delaying IVI in RVO patients led to a short-term decrease in vision and recurrence of ME. However, this initial worsening was not detrimental to patients in terms of long-term BCVA and control of ME, as we observed a full recovery of anatomical structure on OCT and visual function, regardless of the duration of the treatment interruption. These findings provide valuable insights for retina specialists, both in the context of future pandemic-related disruptions and in everyday clinical practice, The treatment delays observed during the Swiss lockdown may reflect those seen in real-life settings, where patients miss injections for various reasons – a challenge frequently encountered by ophthalmologists.

## Supplementary Information

Below is the link to the electronic supplementary material.

Supplementary Material 1: Additional graphic 1. Change in BCVA over time points. BCVA: best corrected visual acuity.

Supplementary Material 2: Additional graphic 2. Change in CST over time points. CST: central subfield thickness.

Supplementary Material 3: Additional graphic 3. Change in IRF and SRF over time points. IRF: intraretinal fluid. SRF: subretinal fluid.

Supplementary Material 4: Additional Table 1. Change in BCVA / CST / IRF / SRF: comparison between subgroups of analysis.BCVA: best corrected visual acuity; CST: central subfield thickness; IRF: intraretinal fluid; IRF+: presence of intraretinal fluid; IRF-: absence of intraretinal fluid; SRF: subretinal fluid; SRF+: presence of subretinal fluid; SRF-: absence of subretinal fluid; NA: not applicable; btw: variation between; RE: right eye; LE: left eye; IQR: Interquartile range

## Data Availability

No datasets were generated or analysed during the current study.

## Abbreviations

AI: Artificial Intelligence
AMD: Age-related macular degeneration
Anti-VEGF: Anti-Vascular Endothelial Growth Factor
BCVA: Best-Corrected Visual Acuity
BRVO: Branch Retinal Vein Occlusion
CNN: convolutional neural network
CRVO: Central Retinal Vein Occlusion
CST: Central Subfield Thickness
DA: Disc Area
DME: Diabetic Macular Edema
DRIL: Disorganization of the inner retinal layers
ETDRS: Early Treatment Diabetic Retinopathy Study
EZ: Ellipsoid zone
IRF: Intraretinal Fluid
IVI: Intravitreal anti-VEGF Injection
ME: Macular Edema
OCT: Optical Coherence Tomography
RVO: Retinal Vein Occlusion
SD-OCT: Spectral-Domain Optical Coherence Tomography
SRF: Subretinal Fluid
T&E: Treat-and-Extend

## References

[1] Song P, Xu Y, Zha M, Zhang Y, Rudan I. Global epidemiology of retinal vein occlusion: a systematic review and meta-analysis of prevalence, incidence, and risk factors. J Glob Health. 2019;9(1):010427. 31131101 10.7189/jogh.09.010427 PMC6513508

[2] Romano F, Lamanna F, Gabrielle PH, Teo KYC, Battaglia Parodi M, Iacono P, et al. Update on retinal vein occlusion. Asia-Pac J Ophthalmol. 2023;12(2):196–210. 10.1097/APO.0000000000000598 36912792

[3] Hayreh SS. Ocular vascular occlusive disorders: natural history of visual outcome. Prog Retin Eye Res. 2014;41:1–25. 24769221 10.1016/j.preteyeres.2014.04.001 PMC4073304

[4] McIntosh RL, Rogers SL, Lim L, Cheung N, Wang JJ, Mitchell P, et al. Natural history of central retinal vein occlusion: an evidence-based systematic review. Ophthalmology. 2010;117(6):1113–e112315. 20430446 10.1016/j.ophtha.2010.01.060

[5] Trichonas G, Kaiser PK. Optical coherence tomography imaging of macular oedema. Br J Ophthalmol. 2014;98(Suppl 2):ii24–29. 24934220 10.1136/bjophthalmol-2014-305305 PMC4208347

[6] Shalchi Z, Mahroo O, Bunce C, Mitry D. Anti-vascular endothelial growth factor for macular oedema secondary to branch retinal vein occlusion. Cochrane Database Syst Rev. 2020;7(7):CD009510. 32633861 10.1002/14651858.CD009510.pub3 PMC7388176

[7] Braithwaite T, Nanji AA, Lindsley K, Greenberg PB. Anti-vascular endothelial growth factor for macular oedema secondary to central retinal vein occlusion. Cochrane Database Syst Rev. 2014;2014(5):CD007325. 24788977 10.1002/14651858.CD007325.pub3 PMC4292843

[8] Korobelnik JF, Holz FG, Roider J, Ogura Y, Simader C, Schmidt-Erfurth U, et al. Intravitreal Aflibercept injection for macular edema resulting from central retinal vein occlusion: One-Year results of the phase 3 GALILEO study. Ophthalmology. 2014;121(1):202–8. 24084497 10.1016/j.ophtha.2013.08.012

[9] Platania CBM, Di Paola L, Leggio GM, Romano GL, Drago F, Salomone S, et al. Molecular features of interaction between VEGFA and anti-angiogenic drugs used in retinal diseases: a computational approach. Front Pharmacol. 2015;6:248. 26578958 10.3389/fphar.2015.00248 PMC4624855

[10] Ferrara N, Damico L, Shams N, Lowman H, Kim R. Development of ranibizumab, an anti-vascular endothelial growth factor antigen binding fragment, as therapy for neovascular age-related macular degeneration. Retina Phila Pa. 2006;26(8):859–70. 10.1097/01.iae.0000242842.14624.e7 17031284

[11] Papadopoulos N, Martin J, Ruan Q, Rafique A, Rosconi MP, Shi E, et al. Binding and neutralization of vascular endothelial growth factor (VEGF) and related ligands by VEGF trap, Ranibizumab and bevacizumab. Angiogenesis. 2012;15(2):171–85. 22302382 10.1007/s10456-011-9249-6 PMC3338918

[12] Nanji K, Khan M, Khalid MF, Xie JS, Sarohia GS, Phillips M, et al. Treat-and-extend regimens of anti-vascular endothelial growth factor therapy for retinal vein occlusions: a systematic review and meta-analysis. Acta Ophthalmol (Copenh). 2022;100(6):e1199–208. 10.1111/aos.15068 34845830

[13] Korobelnik JF, Larsen M, Eter N, Bailey C, Wolf S, Schmelter T, et al. Efficacy and safety of intravitreal Aflibercept Treat-and-Extend for macular edema in central retinal vein occlusion: the CENTERA study. Am J Ophthalmol. 2021;227:106–15. 33556381 10.1016/j.ajo.2021.01.027

[14] Korobelnik JF, Loewenstein A, Eldem B, Joussen AM, Koh A, Lambrou GN, et al. Guidance for anti-VEGF intravitreal injections during the COVID-19 pandemic. Graefes Arch Clin Exp Ophthalmol Albrecht Von Graefes Arch Klin Exp Ophthalmol. 2020;258(6):1149–56. 10.1007/s00417-020-04703-x PMC7179379 32328757

[15] Lim JI, Rachitskaya AV, Hallak JA, Gholami S, Alam MN. Artificial intelligence for retinal diseases. Asia-Pac J Ophthalmol. 2024;13(4):100096. 10.1016/j.apjo.2024.100096 39209215

[16] Apostolopoulos S, De Zanet S, Ciller C, Wolf S, Sznitman R, Pathological OCT. Retinal Layer Segmentation Using Branch Residual U-Shape Networks. In: Descoteaux M, Maier-Hein L, Franz A, Jannin P, Collins DL, Duchesne S, editors. Medical Image Computing and Computer Assisted Intervention– MICCAI 2017 [Internet]. Cham: Springer International Publishing; 2017 [cited 2025 Apr 27]. pp. 294–301. (Lecture Notes in Computer Science; vol. 10435). Available from: https://link.springer.com/10.1007/978-3-319-66179-7_34

[17] Mousavi S, Garjani A, Elwakil A, Brock LP, Dherse A, Forestier E et al. Cohort Builder: A Software Pipeline for Generating Patient Cohorts with Predetermined Baseline Characteristics from Medical Records and Raw Ophthalmic Imaging Data. In: Mantas J, Hasman A, Demiris G, Saranto K, Marschollek M, Arvanitis TN, editors. Studies in Health Technology and Informatics [Internet]. IOS Press; 2024 [cited 2025 Apr 27]. Available from: https://ebooks.iospress.nl10.3233/SHTI240613 10.3233/SHTI240613 39176584

[18] Swiss Federal Council. Ordinance on measures to combat the coronavirus (COVID-19). Available at: https://www.fedlex.admin.ch/eli/cc/2020/141/en

[19] Carnevali A, Giannaccare G, Gatti V, Scuteri G, Randazzo G, Scorcia V. Intravitreal injections during COVID-19 outbreak: Real-world experience from an Italian tertiary referral center. Eur J Ophthalmol. 2021;31(1):10–2. 32967465 10.1177/1120672120962032

[20] Kodjikian L. [How to approach intravitreal injections during this COVID-19 pandemic?]. J Fr Ophtalmol. 2020;43(6):539–40. 32451137 10.1016/j.jfo.2020.04.019 PMC7198140

[21] Mantel I, Mosinska A, Bergin C, Polito MS, Guidotti J, Apostolopoulos S, et al. Automated quantification of pathological fluids in neovascular Age-Related macular degeneration, and its repeatability using deep learning. Transl Vis Sci Technol. 2021;10(4):17. 34003996 10.1167/tvst.10.4.17 PMC8083067

[22] Navarrete A, Vofo B, Matos K, Rivera A, Levy J. The detrimental effects of delayed intravitreal anti-VEGF therapy for treating retinal pathology: lessons from a forced test-case. Graefes Arch Clin Exp Ophthalmol Albrecht Von Graefes Arch Klin Exp Ophthalmol. 2022;260(7):2201–8. 10.1007/s00417-021-05549-7 PMC8739509 34994841

[23] Montesel A, Gigon A, Giacuzzo C, Mantel I, Eandi CM. TREATMENT DEFERRAL DURING COVID-19 LOCKDOWN: functional and anatomical impact on patients with neovascular Age-Related macular degeneration. Retina Phila Pa. 2022;42(4):634–42. 10.1097/IAE.0000000000003369 PMC8946588 34907122

[24] Song W, Kanyo E, Bastian R, Singh RP, Rachitskaya AV. Visual acuity in patients requiring intravitreal injections: Short-Term and Long-Term effects of delay in care. J Vitreoretin Dis. 2023;7(1):20–6. 37008399 10.1177/24741264221136637 PMC9954165

[25] Koss MJ, Pfister M, Rothweiler F, Michaelis M, Cinatl J, Schubert R et al. Comparison of cytokine levels from undiluted vitreous of untreated patients with retinal vein occlusion. Acta Ophthalmol (Copenh) [Internet]. 2012 Mar [cited 2025 Jul 9];90(2). Available from: https://onlinelibrary.wiley.com/doi/10.1111/j.1755-3768.2011.02292.x 10.1111/j.1755-3768.2011.02292.x 22066978

[26] Noma H, Funatsu H, Yamasaki M, Tsukamoto H, Mimura T, Sone T, et al. Aqueous humour levels of cytokines are correlated to vitreous levels and severity of macular oedema in branch retinal vein occlusion. Eye Lond Engl. 2008;22(1):42–8. 10.1038/sj.eye.6702498 16826241

[27] Noma H, Funatsu H, Mimura T, Harino S, Hori S. Vitreous levels of interleukin-6 and vascular endothelial growth factor in macular edema with central retinal vein occlusion. Ophthalmology. 2009;116(1):87–93. 19118700 10.1016/j.ophtha.2008.09.034

[28] Campochiaro PA, Sophie R, Pearlman J, Brown DM, Boyer DS, Heier JS, et al. Long-term outcomes in patients with retinal vein occlusion treated with ranibizumab: the RETAIN study. Ophthalmology. 2014;121(1):209–19. 24112944 10.1016/j.ophtha.2013.08.038

[29] Khan MA, Mallika V, Joshi D. Comparison of immediate versus deferred intravitreal bevacizumab in macular oedema due to branch retinal vein occlusion: a pilot study. Int Ophthalmol. 2018;38(3):943–9. 28432581 10.1007/s10792-017-0538-y

[30] Epstein DL, Algvere PV, Von Wendt G, Seregard S, Kvanta A. Benefit from bevacizumab for macular edema in central retinal vein occlusion: Twelve-Month results of a prospective, randomized study. Ophthalmology. 2012;119(12):2587–91. 22902212 10.1016/j.ophtha.2012.06.037

[31] Larsen M, Waldstein SM, Boscia F, Gerding H, Monés J, Tadayoni R, et al. Individualized Ranibizumab regimen driven by stabilization criteria for central retinal vein occlusion: Twelve-Month results of the CRYSTAL study. Ophthalmology. 2016;123(5):1101–11. 26896124 10.1016/j.ophtha.2016.01.011

[32] Brown DM, Heier JS, Clark WL, Boyer DS, Vitti R, Berliner AJ, et al. Intravitreal Aflibercept injection for macular edema secondary to central retinal vein occlusion: 1-year results from the phase 3 COPERNICUS study. Am J Ophthalmol. 2013;155(3):429–e4377. 23218699 10.1016/j.ajo.2012.09.026

[33] Nicholson L, Talks SJ, Amoaku W, Talks K, Sivaprasad S. Retinal vein occlusion (RVO) guideline: executive summary. Eye. 2022;36(5):909–12. 35301458 10.1038/s41433-022-02007-4 PMC9046155

[34] Agata C, Aoki S, Kitamoto K, Azuma K, Fujino R, Inoue T, et al. Time to initiate anti-vascular endothelial growth factor therapy and visual outcome in central retinal vein occlusion. Sci Rep. 2024;14(1):16974. 39043891 10.1038/s41598-024-67925-7 PMC11266534

[35] Im JHB, Jin YP, Chow R, Dharia RS, Yan P. Delayed anti-VEGF injections during the COVID-19 pandemic and changes in visual acuity in patients with three common retinal diseases: A systematic review and meta-analysis. Surv Ophthalmol. 2022;67(6):1593–602. 35970234 10.1016/j.survophthal.2022.08.002 PMC9374495

[36] Liu JC, Alsaloum P, Iyer AI, Kaiser PM, Singh RP. Consequences of anti-vascular endothelial growth factor treatment lapse in patients with retinal vein occlusion. Eye. 2023;37(3):453–8. 35132210 10.1038/s41433-022-01960-4 PMC9905085

[37] Abraham JR, Boss J, Babiuch AS, Singh RP, Srivastava S, Reese J, et al. Longitudinal assessment of ellipsoid zone mapping parameters in retinal venous occlusive disease with associated macular edema. J Vitreoretin Dis. 2021;5(1):40–5. 37009581 10.1177/2474126420943418 PMC9976050

[38] Lee MW, Jun JH, Seong HJ. Longitudinal changes in each retinal layer thickness in patients with non-ischemic central retinal vein occlusion. Eye Vis. 2024;11(1):29. 10.1186/s40662-024-00397-y PMC11293173 39085961

[39] Gallardo M, Munk MR, Kurmann T, De Zanet S, Mosinska A, Karagoz IK, et al. Machine learning can predict Anti-VEGF treatment demand in a Treat-and-Extend regimen for patients with neovascular AMD, DME, and RVO associated macular edema. Ophthalmol Retina. 2021;5(7):604–24. 33971352 10.1016/j.oret.2021.05.002

[40] Jee D, Park S, Kwon Jwoo. Subretinal fluid in macular edema secondary to branch retinal vein occlusion. Sci Rep. 2024;14(1):13623. 38871805 10.1038/s41598-024-64047-y PMC11176314

[41] Sasajima H, Zako M, Maeda R, Murotani K, Ishida H, Ueta Y. Foveal intraretinal fluid localization affects the visual prognosis of branch retinal vein occlusion. J Clin Med. 2022;11(12):3540. 35743609 10.3390/jcm11123540 PMC9224585

