# Supplementary figures and images for: Short and long-term impact of intravitreal anti-VEGF therapy interruption in retinal vein occlusion during the COVID-19 pandemic: functional outcomes and AI-based fluid analysis of macular edema
Source: Int J Retina Vitreous. 2025 Aug 5;11:92. doi: 10.1186/s40942-025-00717-x (PMC12326874; doi:10.1186/s40942-025-00717-x)

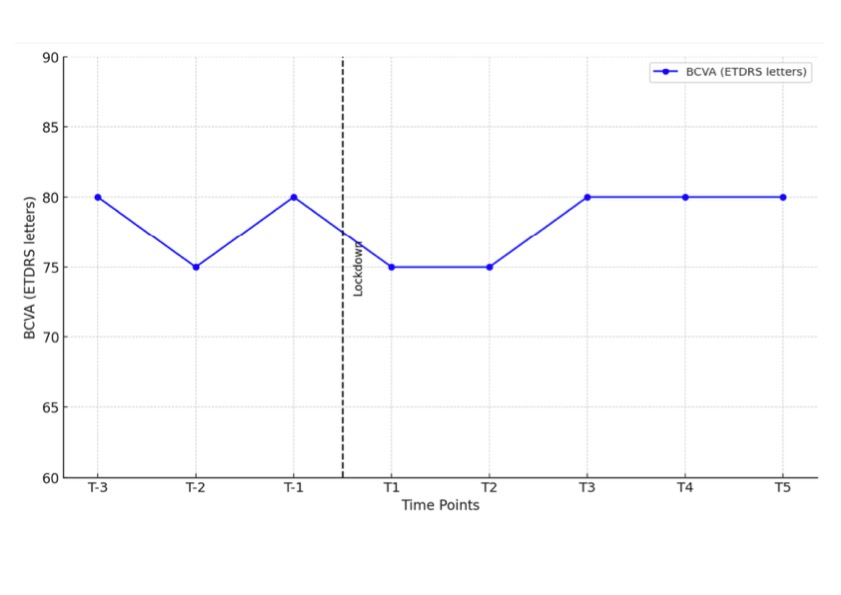

Supplement: Supplementary file 1 — Supplementary Material 1: Additional graphic 1. Change in BCVA over time points. BCVA: best corrected visual acuity. [file 40942_2025_717_MOESM1_ESM.jpg]

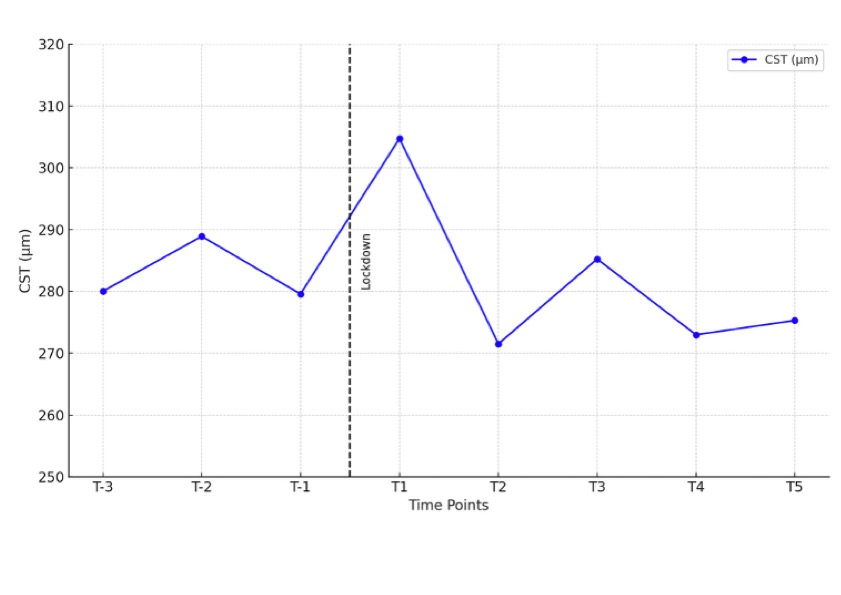

Supplement: Supplementary file 2 — Supplementary Material 2: Additional graphic 2. Change in CST over time points. CST: central subfield thickness. [file 40942_2025_717_MOESM2_ESM.jpg]

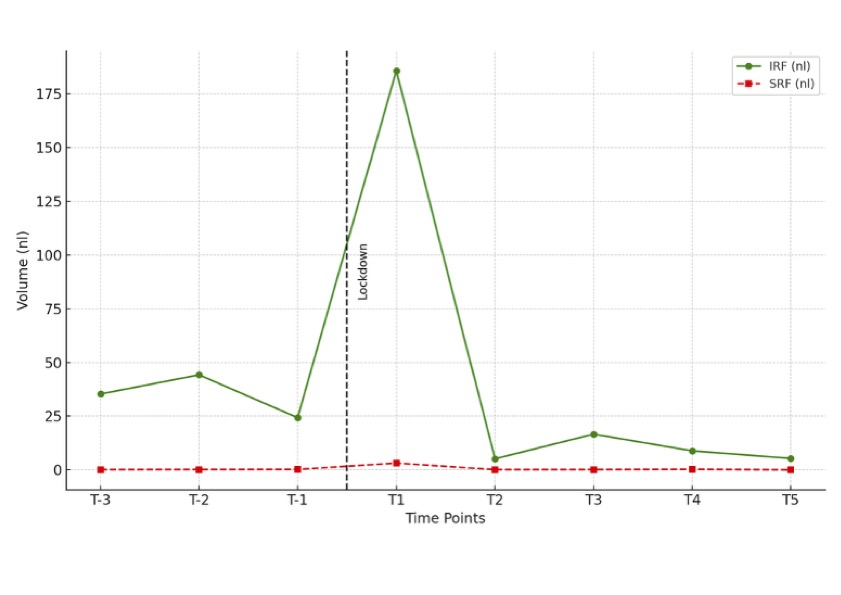

Supplement: Supplementary file 3 — Supplementary Material 3: Additional graphic 3. Change in IRF and SRF over time points. IRF: intraretinal fluid. SRF: subretinal fluid. [file 40942_2025_717_MOESM3_ESM.jpg]
